# Supplementary material for: A Possibility to Use Selected Crop Post-Extraction Wastes to Improve the Composition of Cultivated Mushroom Pleurotus citrinopileatus
Source: J Fungi (Basel). 2021 Oct 22;7(11):894. doi: 10.3390/jof7110894 (PMC8625471; doi:10.3390/jof7110894)
Supplement: Supplementary file 1 [file jof-07-00894-s001.zip › jof-1388301-supplementary.pdf]

Table S1. Validation data for elemental analysis by ICP-OES

	wavelength	DL	range	uncertainty		wavelength	DL	range	uncertainty
	nm	mg kg^{-1}	mg kg^{-1}	%		nm	mg kg^{-1}	mg kg^{-1}	%
**Ag**	328.068	0.0086	DL-200	10.0	**Mo**	202.032	0.044	DL-100	3.1
**Al**	394.401	x	200-4000	6.5	**Na**	588.995	0.26	DL-1000	6.7
**Al**	396.152	0.0053	DL-200	2.9	**Na**	589.592	x	1000-10000	3.4
**As**	188.980	0.012	DL-100	14.6	**Nb**	313.078	0.0067	DL-100	5.8
**Au**	197.742	0.0092	DL-100	18.0	**Nd**	406.108	0.012	DL-100	6.2
**B**	249.772	0.047	DL-200	3.4	**Ni**	231.604	0.0092	DL-200	8.0
**Ba**	455.403	0.002	DL-200	9.0	**Os**	225.585	0.021	DL-100	9.5
**Be**	313.042	0.026	DL-100	12.0	**Pb**	220.353	0.023	DL-200	4.2
**Bi**	223.061	0.065	DL-200	4.1	**Pd**	340.458	0.025	DL-100	9.0
**Ca**	315.887	x	2000-10000	6.4	**Pr**	417.939	0.032	DL-100	9.8
**Ca**	422.673	0.036	DL-2000	2.8	**Pt**	203.646	0.021	DL-100	29.8
**Cd**	214.439	0.0026	DL-200	10.1	**Rb**	780.026	0.045	DL-100	10.1
**Ce**	446.021	0.023	DL-100	13.6	**Re**	197.248	0.032	DL-100	5.0
**Co**	238.892	0.0029	DL-200	1.6	**Rh**	343.488	0.035	DL-100	13.3
**Cr**	267.716	0.0033	DL-200	4.0	**Ru**	240.272	0.021	DL-100	9.4
**Cs**	697.327	0.02	DL-1000	12.1	**S**	181.972	0.44	DL-1000	15.7
**Cu**	327.395	0.0027	DL-200	9.6	**Sb**	206.834	0.012	DL-100	14.0
**Dy**	364.540	0.023	DL-100	6.7	**Sc**	361.383	0.024	DL-100	3.0
**Er**	349.910	0.018	DL-100	7.9	**Se**	196.026	0.011	DL-100	16.4
**Eu**	420.504	0.034	DL-100	3.0	**Si**	288.158	0.59	DL-1000	19.2
**Fe**	238.204	0.0084	DL-1000	1.6	**Sm**	442.434	0.026	DL-100	9.8
**Fe**	261.382	x	100-4000	0.89	**Sn**	283.998	0.067	DL-100	12.3
**Ga**	294.363	0.0097	DL-200	3.4	**Sr**	460.733	0.0092	DL-200	5.3
**Gd**	342.246	0.034	DL-100	6.1	**Ta**	268.517	0.012	DL-100	6.5
**Ge**	209.426	0.039	DL-100	2.2	**Tb**	350.914	0.027	DL-100	7.3
**Hf**	264.141	0.012	DL-100	5.8	**Te**	214.282	0.011	DL-100	5.5
**Hg**	194.164	0.023	DL-100	14.6	**Th**	283.730	0.015	DL-100	4.1
**Ho**	348.484	0.031	DL-100	11.9	**Ti**	336.122	0.021	DL-100	5.7
**In**	230.606	0.025	DL-200	8.0	**Tl**	190.794	0.024	DL-200	7.3
**Ir**	205.116	0.0097	DL-100	6.5	**Tm**	336.261	0.031	DL-100	7.6
**K**	766.491	0.34	DL-1000	1.3	**U**	385.957	0.011	DL-100	5.6
**K**	769.897	x	1000-10000	0.53	**V**	292.401	0.018	DL-100	6.2
**La**	398.852	0.0086	DL-100	6.8	**W**	207.912	0.027	DL-1000	12.3
**Li**	670.783	0.0044	DL-200	16.6	**Y**	361.104	0.031	DL-100	6.2
**Lu**	307.760	0.031	DL-100	2.9	**Yb**	328.937	0.014	DL-100	3.8
**Mg**	279.553	0.008	DL-500	9.7	**Zn**	213.857	0.0022	DL-200	3.5
**Mg**	285.213	x	500-5000	4.5	**Zr**	343.823	0.018	DL-100	6.5
**Mn**	257.610	0.0021	DL-200	1.8					
